# Supplementary material for: ‘Rewritable’ and ‘liquid-specific’ recognizable wettability pattern
Source: Nat Commun. 2024 Jul 11;15:5838. doi: 10.1038/s41467-024-49807-8 (PMC11239882; doi:10.1038/s41467-024-49807-8)
Supplement: Supplementary file 1 — Supplementary Information [file 41467_2024_49807_MOESM1_ESM.pdf]

‘Rewritable’ and ‘Liquid-specific’ recognizable wettability pattern

Manideepa Dhar[1], Debasmita Sarkar[1], Avijit Das[1], SK Asif Rahaman[1], Dibyendu Ghosh[1] and Uttam Manna[1,2,3*]

[1] Department of Chemistry, Indian Institute of Technology-Guwahati, Assam, 781039 India,
[2] Centre for Nanotechnology, Indian Institute of Technology-Guwahati, Assam 781039 India,
[3] Jyoti and Bhupat Mehta School of Health Science & Technology, Indian Institute of Technology-Guwahati, Assam, 781039 India,
* email: umanna@iitg.ac.in

Figure number	Description of Figure	Page number
Supplementary Figure 1	NMR spectra of different comb-like copolymers ($PODAMA_{1/2/3/4}$).	S-3
Supplementary Figure 2	FTIR spectra of glucamine modified PODAMA.	S-4
Supplementary Figure 3	Characterization of hydrophilic porous polymeric coating	S-5
Supplementary Figure 4	Wettability study and characterization of $RPIC_1$, $RPIC_2$, $RPIC_3$ and $RPIC_4$.	S-6
Supplementary Figure 5	AFM images of $RPIC_1$, $RPIC_2$, $RPIC_3$ and $RPIC_4$.	S-7
Supplementary Figure 6	FTIR characterization of PLAMA and wettability study on coating derived from PLAMA.	S-8
Supplementary Figure 7	Wetting properties of $RPIC_2$ towards different liquids.	S-9
Supplementary Figure 8	Solid slippery property of $RPIC_2$ towards different liquids.	S-10
Supplementary Figure 9	FTIR characterization of PDAMA and wetting properties of PDAMA based coating towards different liquids.	S-11
Supplementary Figure 10	Slippery property of PDAMA based coating towards different liquids.	S-12
Supplementary Figure 11	Optical transparency of $RPIC_1$, $RPIC_2$, $RPIC_3$ and $RPIC_4$.	S-13
Supplementary Figure 12	FESEM image of $RPIC_2$.	S-14
Supplementary Figure 13	DSC curve of $PODAMA_2$ before and after glucamine modification.	S-16
Supplementary Figure 14	Physical scratch healing study.	S-17

Supplementary Figure 15	Change in slippery property of Glu modified $RPIC_2$ against water and ethanol.	S-18
Supplementary Figure 16	Selective sacrifice of slippery property of Glu-modified $RPIC_2$ against liquids having surface tension < 30 mN m^{-1}.	S-19
Supplementary Figure 17	Sliding of liquids having surface tension > 30 mN m^{-1} on $RPIC_2$ before and after glucamine modification.	S-20
Supplementary Figure 18	Selective alteration of slippery property of Glu-modified $RPIC_2$ against different liquids.	S-21
Supplementary Figure 19	Spillage of liquid droplets having surface tension (γ) < 30 mN m^{-1} over a range of tilting angles.	S-22
Supplementary Figure 20	Spillage of liquid droplets having surface tension (γ) < 30 mN m^{-1} irrespective of their volume.	S-23
Supplementary Figure 21	Spillage of organic liquids and oils on glucamine modified $RPIC_{PDAMA}$.	S-24
Supplementary Figure 22	Change in roughness and surface free energy of $RPIC_{PDAMA}$ before and after glucamine modification.	S-25
Supplementary Figure 23	Restoring native wettability of Glu-modified $RPIC_2$.	S-16
Supplementary Figure 24	Change in thickness of $RPIC_2$ by varying loading amount of infused polymer.	S-26
Supplementary Figure 25	Alteration of ethanol contact angle and surface free energy during multiple cycles of glucamine modification and heat treatment for different sets of $RPIC_2$	S-27
Supplementary Figure 26	Liquid droplet sorting based on their surface tension.	S-28
Supplementary Figure 27	Selective spillage of ethanol at low tilting angle.	S-29
Supplementary Figure 28	Separation of water-hexanol droplets mixture and their NMR characterizations.	S-30

Supplementary Note 1. The synthesized co-polymers ($PODAMA_{1/2/3/4}$) were characterized using ^{1}H NMR spectroscopy. It was observed that MA content gradually increased from $PODAMA_1$ to $PODAMA_4$.

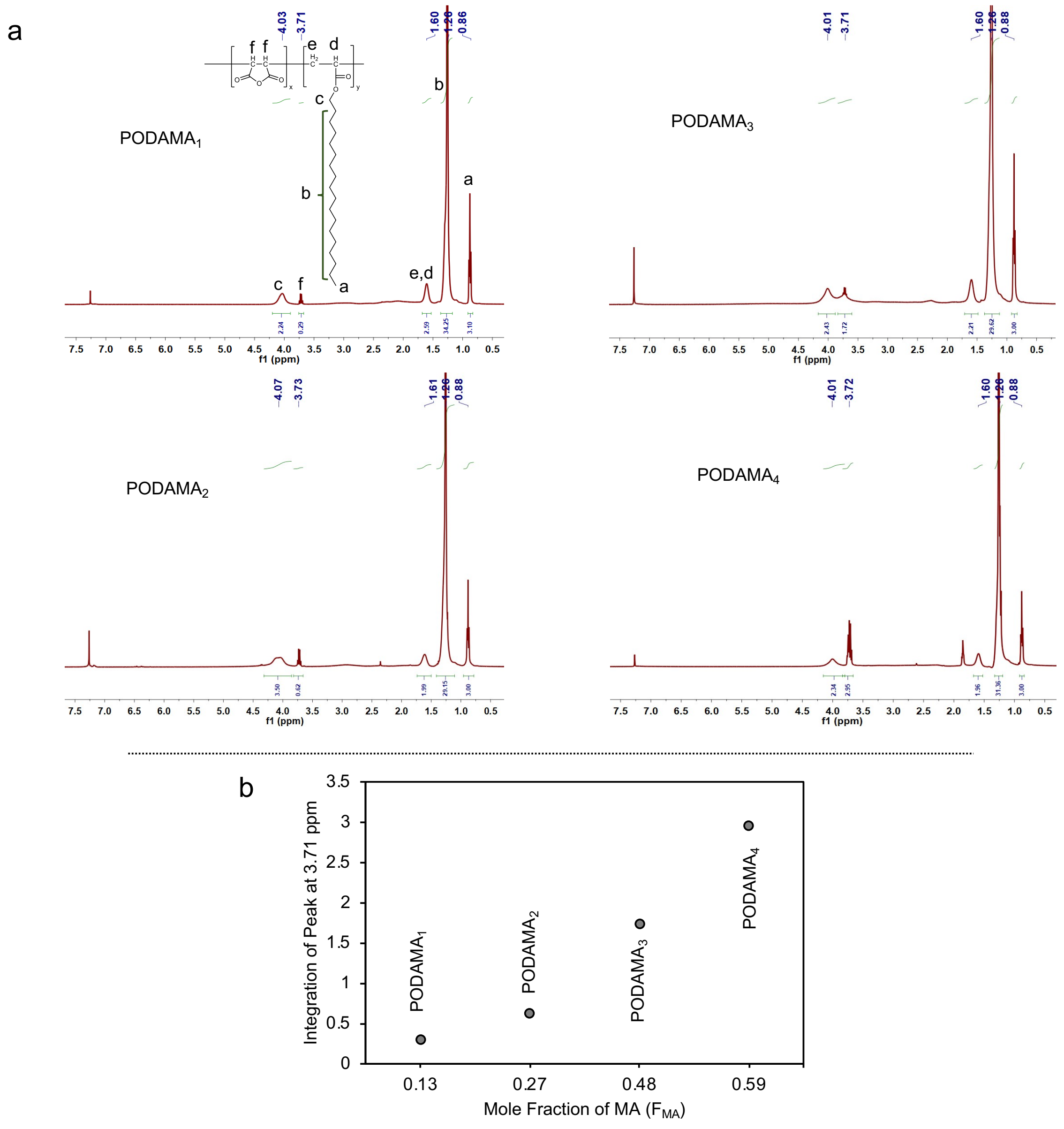

Supplementary Figure 1. Characterization of different co-polymers with ^{1}H NMR spectroscopy. a ^{1}H NMR spectra of $PODAMA_{1/2/3/4}$, where the content of MA moiety has gradually varied in the prepared polymers. **b** Plot accounting for the change in integration area of the peak at 3.71 ppm with the change in MA mole fraction in the prepared polymers ($PODAMA_{1/2/3/4}$). Source data are provided as a Source Data file.

Supplementary Note 2. FTIR spectroscopy was used to characterize the post-chemical modification of PODAMA with glucamine. The appearance of two new peaks at 1641 cm^{-1} and 1564 cm^{-1}, corresponding to amide-I and amide-II indicated the ring opening reaction between PODAMA and glucamine. Also, the depletion peaks at 1846 cm^{-1} and 1782 cm^{-1} corresponding to the carbonyl group of the maleic anhydride ring revalidated the post-covalent modification of PODAMA with glucamine.

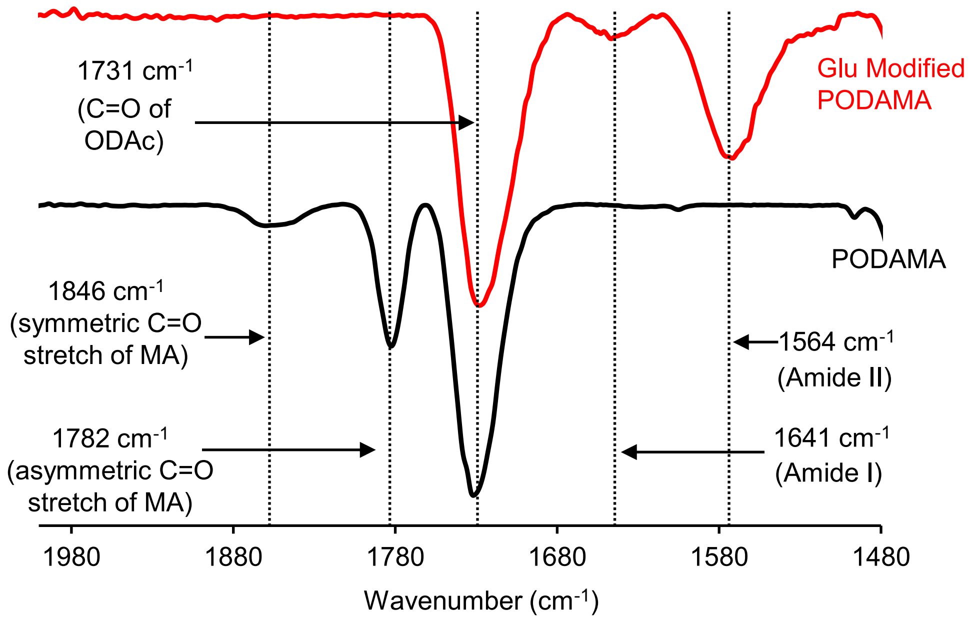

Supplementary Figure 2. Characterization of post-chemical modification reaction of PODAMA. FTIR spectra of PODAMA polymer before (black) and after (red) glucamine (Glu) modification. Source data are provided as a Source Data file.

Supplementary Note 3. To derive RPIC, a hydrophilic porous matrix was developed by spray depositing a reaction mixture solution of BPEI and 5-Acl on glass substrate. This porous matrix inherently remained incapable of sliding beaded droplet of water and ethanol.

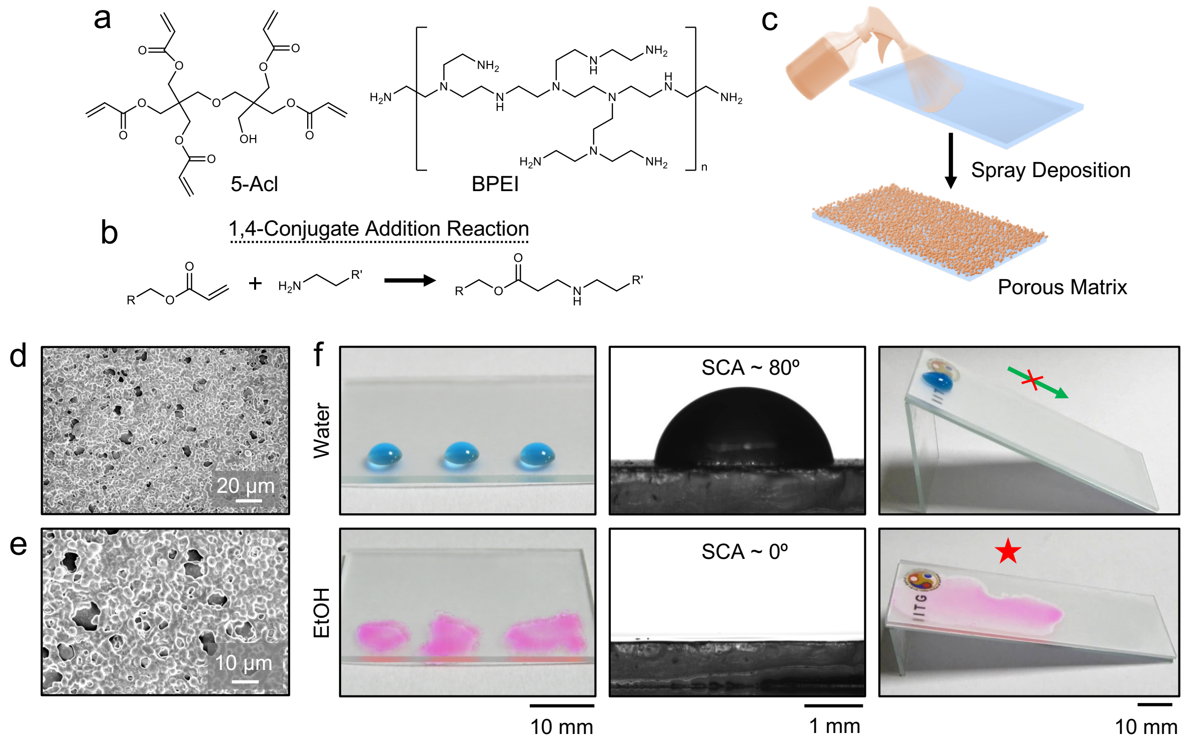

Supplementary Figure 3. Surface morphology and wettability characterization of porous matrix. a Chemical structure of dipentaerythritol penta-acrylate (5-Acl) and branched polyethylenamine (BPEI). **b** Schematic illustration of 1,4-conjugate addition reaction between amine and acrylate functional groups. **c** Schematic representing the fabrication of porous matrix through spray deposition of a reaction mixture solution containing 5-Acl and BPEl. **d,e** Low (d) and high (e) magnifications FESEM images of the porous matrix. **f** Photographs and contact angle images depicting the pinning and spillage of water and ethanol droplets on porous polymeric matrix.

Supplementary Note 4. $RPIC_{1/2/3/4}$ displayed distinct liquid wettability and sliding behavior, which was characterized by capturing digital images and measuring contact and sliding angle images. The elevated content of MA moiety compromised the sliding behavior of the prepared coatings.

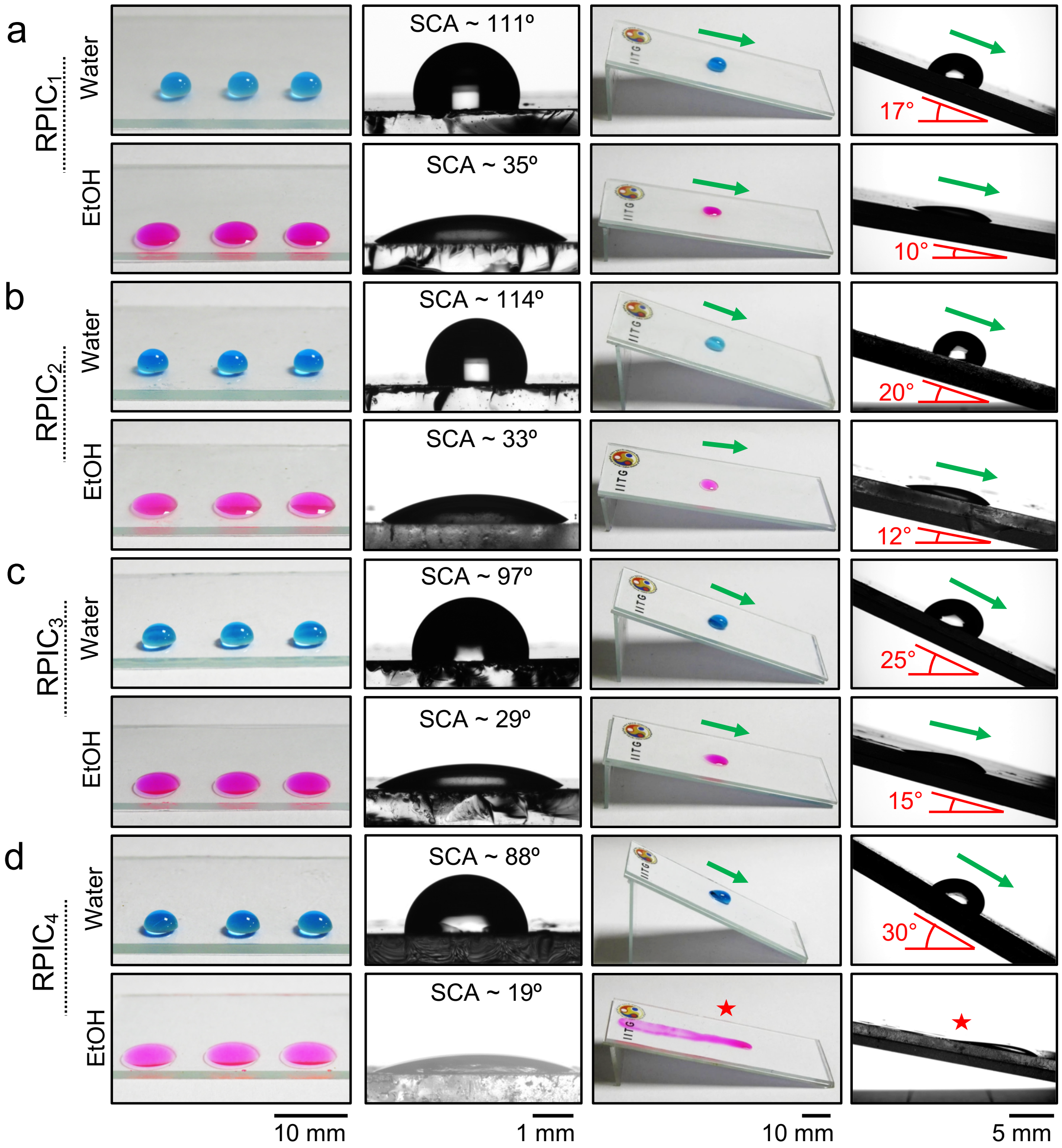

Supplementary Figure 4. Characterization of wettability and sliding behaviour of $RPIC_{1/2/3/4}$. Digital and contact angle images depicting the beading and sliding or spillage of water and ethanol droplets on $RPIC_1$ (a), $RPIC_2$ (b), $RPIC_3$ (c), and $RPIC_4$ (d). Star mark indicates spillage of beaded droplets on solid surface.

Supplementary Note 5. The AFM investigation on the surface morphologies of $RPIC_{1/2/3/4}$ revealed the gradual depletion of roughness with increasing the MA content in the infused polymers ($PODAMA_{1/2/3/4}$) layer in the prepared coatings.

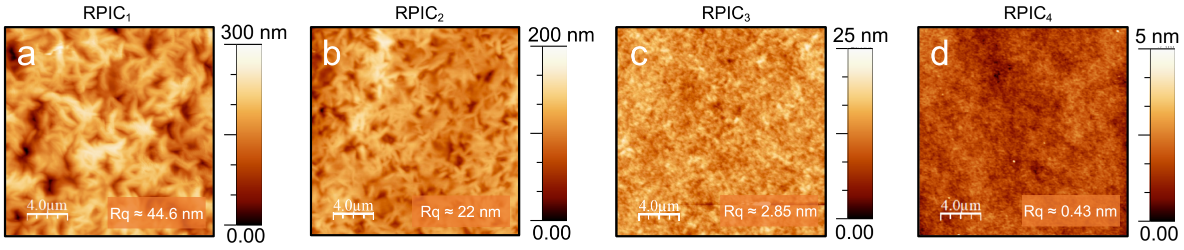

Supplementary Figure 5. Characterization of surface morphologies of $RPIC_{1/2/3/4}$. Atomic force microscope (AFM) images of $RPIC_1$ (a), $RPIC_2$ (b), $RPIC_3$ (c) and $RPIC_4$(d).

Supplementary Note 6. FTIR characterization of PLAMA having shorter hydrocarbon chain compare to PODAMA revealed the existence of residual reactivity. But, the PLAMA infused coating failed to slide beaded droplets of both water (high surface tension liquid) and ethanol (low surface tension liquid). Antiwetting property of PLAMA infused coating was characterized by capturing digital images and measuring contact and sliding angles.

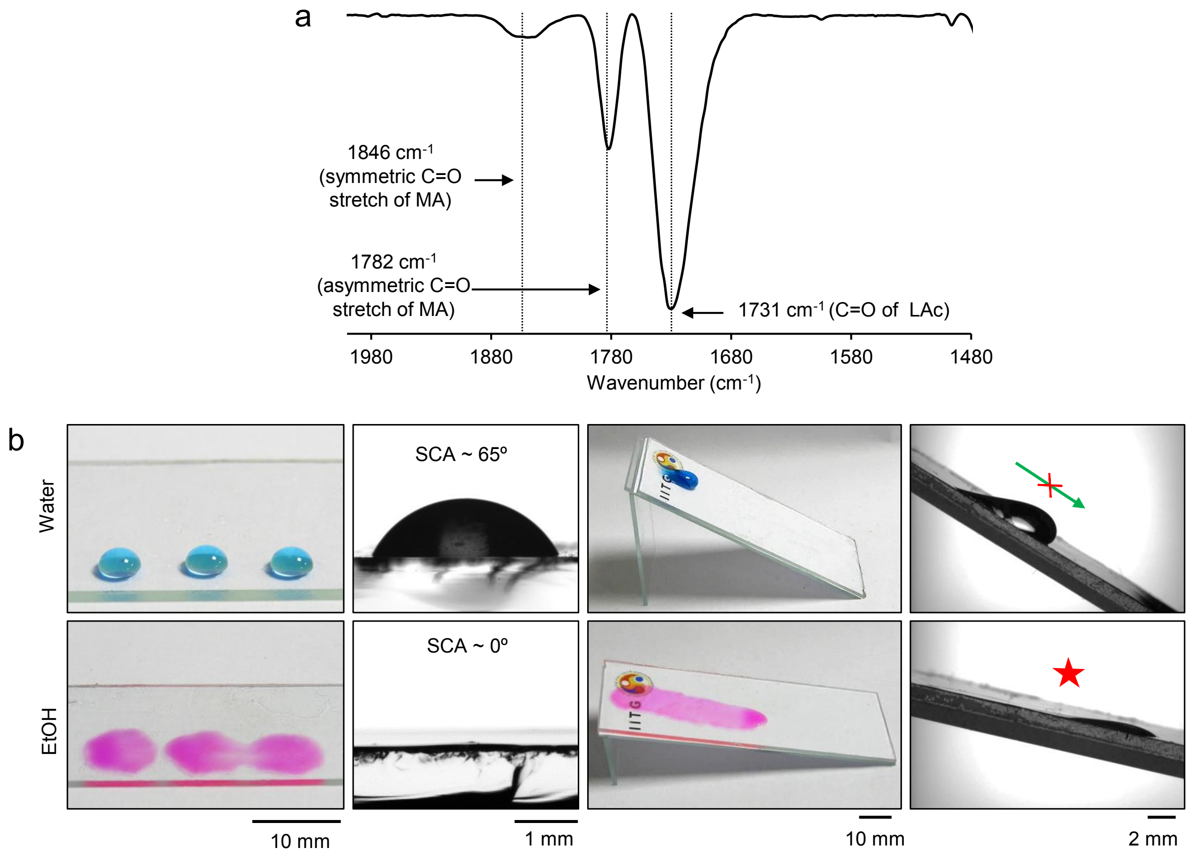

Supplementary Figure 6: **FTIR Characterization of PLAMA and anti-wetting study of PLAMA infused coating**. **a** FTIR spectra of polylauryl acrylate maleic anhydride (PLAMA). **b** Digital and contact angle images depicting the beading, pinning and spillage of water and ethanol droplets on PLAMA-infused coating. Star mark indicates spillage of beaded droplets on solid surface. Source data are provided as a Source Data file.

Supplementary Note 7. Droplets of wide range of non-polar and polar liquids beaded on PODAMA2 infused coating (RPIC2) without arbitrary spillages. The liquid wettability of the coating was characterized by capturing digital images and measuring static contact angles.

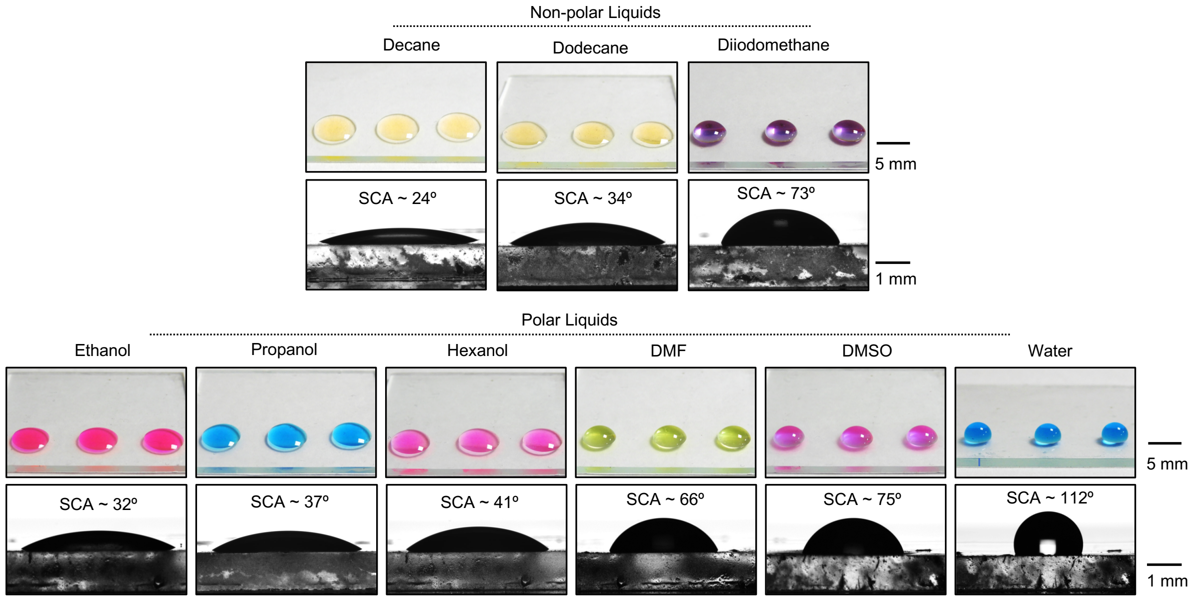

Supplementary Figure 7. Characterization of wettability of PODAMA2 infused coating (RPIC2). Digital and static contact angle (SCA) images depicting the beading of different liquids on solid slippery coating.

Supplementary Note 8. PODAMA2 infused coating (RPIC2) remained capable of sliding a wide range of non-polar and polar liquids. The liquid droplet sliding behaviour on the RPIC2 was characterized by capturing digital images and measuring sliding angles.

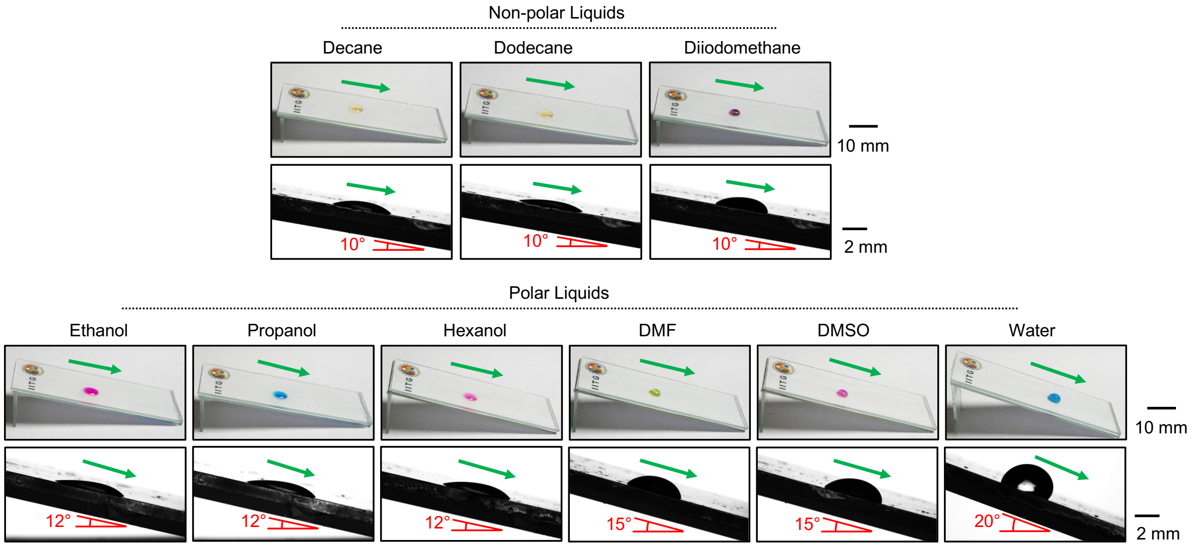

Supplementary Figure 8. Characterization of anti-wetting property of PODAMA2 infused coating (RPIC2). Digital and contact angle images depicting the sliding of different polar and non-polar liquids on the prepared solid slippery coating.

Supplementary Note 9. FTIR characterization of PDAMA validated the existence of residual reactivity. Droplets of a wide range of non-polar, polar liquids and oils beaded on the PDAMA infused coating without arbitrary spillage. The liquid wettability of PDAMA infused coating was characterized by capturing digital images and measuring static contact angles.

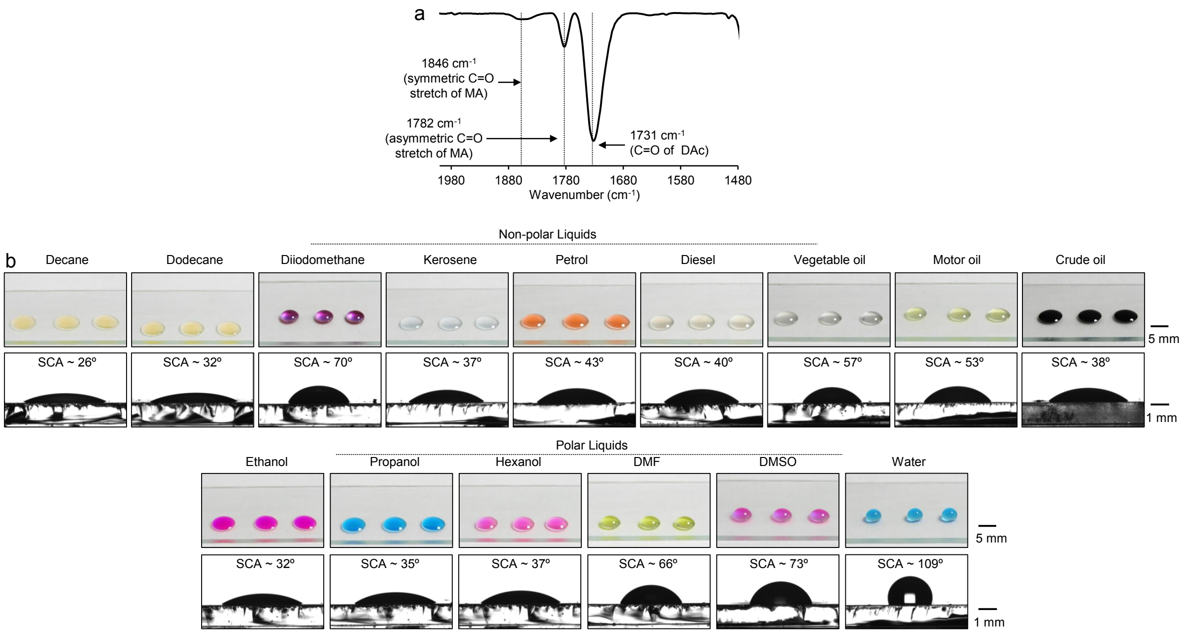

Supplementary Figure 9. Characterization of PDAMA and wettability study on PDAMA infused coating. a FTIR spectra of polydocosyl acrylate maleic anhydride (PDAMA). **b** Photographs depicting the beading of different liquids and oils on solid slippery coating—derived from PDAMA. Source data are provided as a Source Data file.

Supplementary Note 10. PDAMA infused coating displayed slippery property towards a wide range of non-polar, polar liquids and oils. The sliding behaviour of beaded liquid droplets on PDAMA infused coating was characterized by capturing digital images and measuring sliding angles.

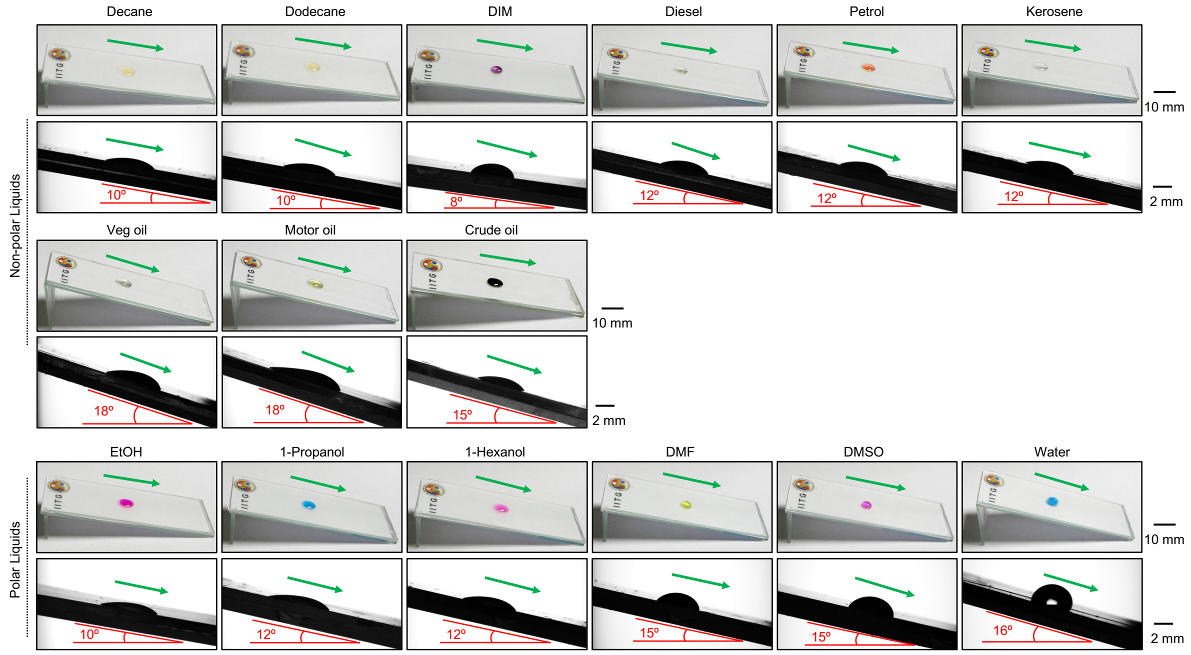

Supplementary Figure 10. Slippery property of PDAMA-infused solid slippery coating. Digital and contact angle images depicting the sliding of different non-polar and polar liquids and oils on PDAMA-infused solid slippery coating.

Supplementary Note 11. Prepared coatings ($RPIC_{1/2/3/4}$) derived from synthesized polymers ($PODAMA_{1/2/3/4}$) displayed difference in optical transparency. An improvement of optical transparency was noted on increasing the content of maleic anhydride moiety in the prepared polymer. UV-Vis spectroscopy was used to characterize the optical transparency of the prepared coatings ($RPIC_{1/2/3/4}$).

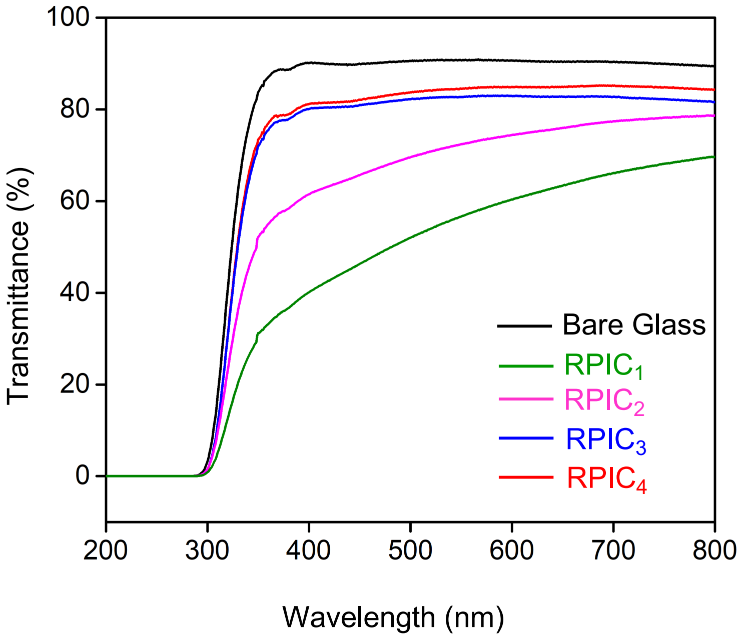

Supplementary Figure 11. Characterization of optical transparency of $RPIC_{1/2/3/4}$. Accounting optical transparency of $RPIC_1$, $RPIC_2$, $RPIC_3$ and $RPIC_4$. Source data are provided as a Source Data file.

Supplementary Note 12. Surface morphology of $RPIC_2$ was examined with FESEM imaging. $RPIC_2$ appeared as a smooth interface.

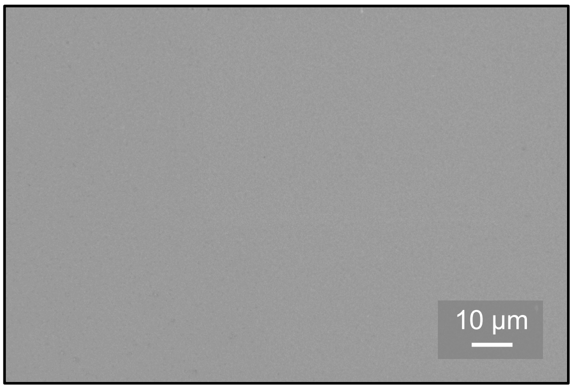

Supplementary Figure 12. Surface morphology characterization of $RPIC_2$. Field emission scanning electron microscope (FESEM) image of $RPIC_2$.

Supplementary Note 13. Phase transition behaviour of $PODAMA_2$ and Glu modified $PODAMA_2$ was characterized using DSC. Both polymers melted upon heating and again solidified upon cooling down to room temperature. A slight depletion of phase transition temperature (from 54.6 °C to 51.9 °C) was noted after post modification of $PODAMA_2$ with glucamine.

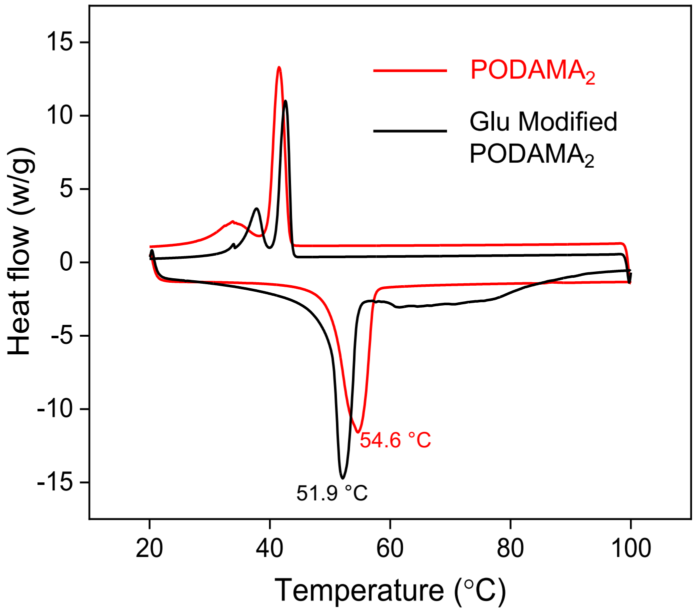

Supplementary Figure 13. **Characterization of phase transition behaviour of $PODAMA_2$.** Differential scanning calorimetric (DSC) study showing the phase transition behaviour of $PODAMA_2$ and glucamine (Glu) modified $PODAMA_2$. Source data are provided as a Source Data file.

Supplementary Note 14. $RPIC_2$ displayed healing of physical damages and compromised anti-wetting properties due to the temperature assisted phase transition behaviour of the infused polymer. This phenomenon was characterized by capturing digital images.

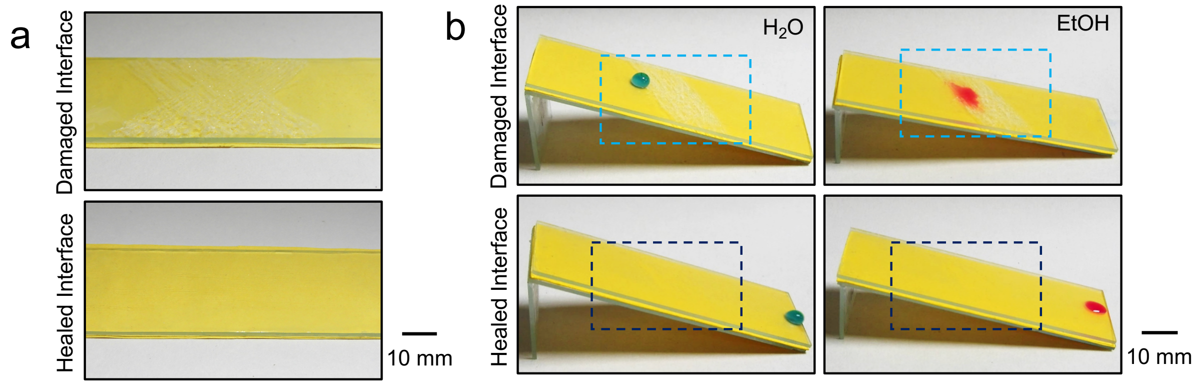

Supplementary Figure 14. Healing of physical damage and compromised anti-wetting property. a Photographs of physically damaged and healed $RPIC_2$ interface. **b** Photographs depicting the pinning and sliding of beaded water and ethanol droplets on physically damaged and healed $RPIC_2$ interface.

Supplementary Note 15. The anti-wetting property of $RPIC_2$ was significantly and selectively altered for low surface tension liquids on incurring the glucamine modification. While, the gluicamine modified coating absolutely failed to slide beaded droplet of low surface tension liquid, i.e. ethanol, the beaded droplet of water (high surface tension liquid,) continued to slide on the tilted interface.

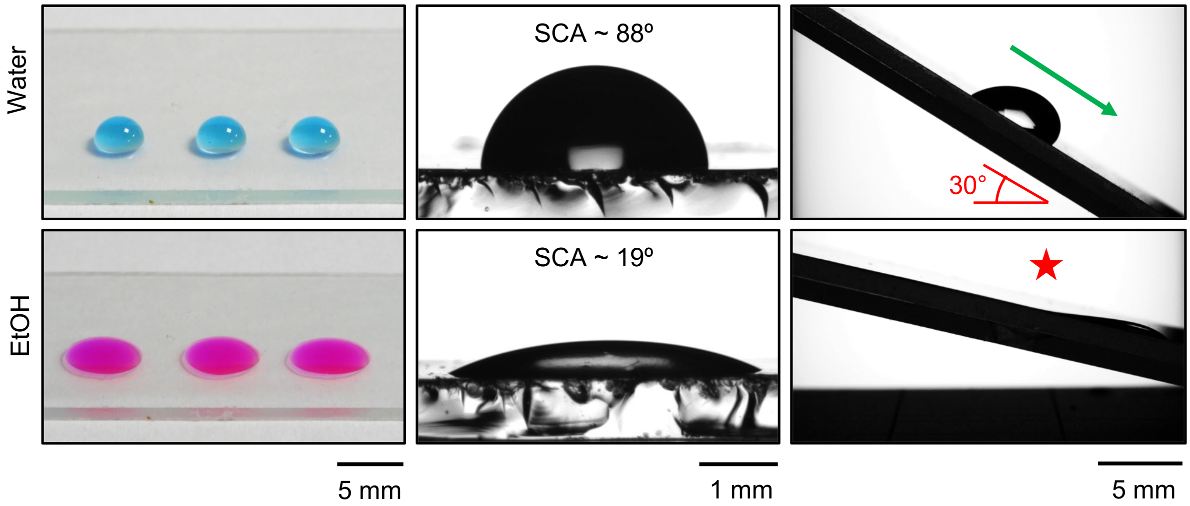

Supplementary Figure 15. Characterization of anti-wetting property of glucamine-modified $RPIC_2$. Photographs and contact angle images showing the beading, sliding or spillage of water and ethanol droplets on glucamine-modified $RPIC_2$. Star mark indicates spillage of beaded droplets on solid surface.

Supplementary Note 16. Glucamine modified $RPIC_2$ failed to slide beaded droplets of liquids having surface tension < 30 mN m^{-1}. However, the droplets of same liquids (surface tension < 30 mN m^{-1}) effortlessly slide on the tilted interface of unmodified $RPIC_2$.

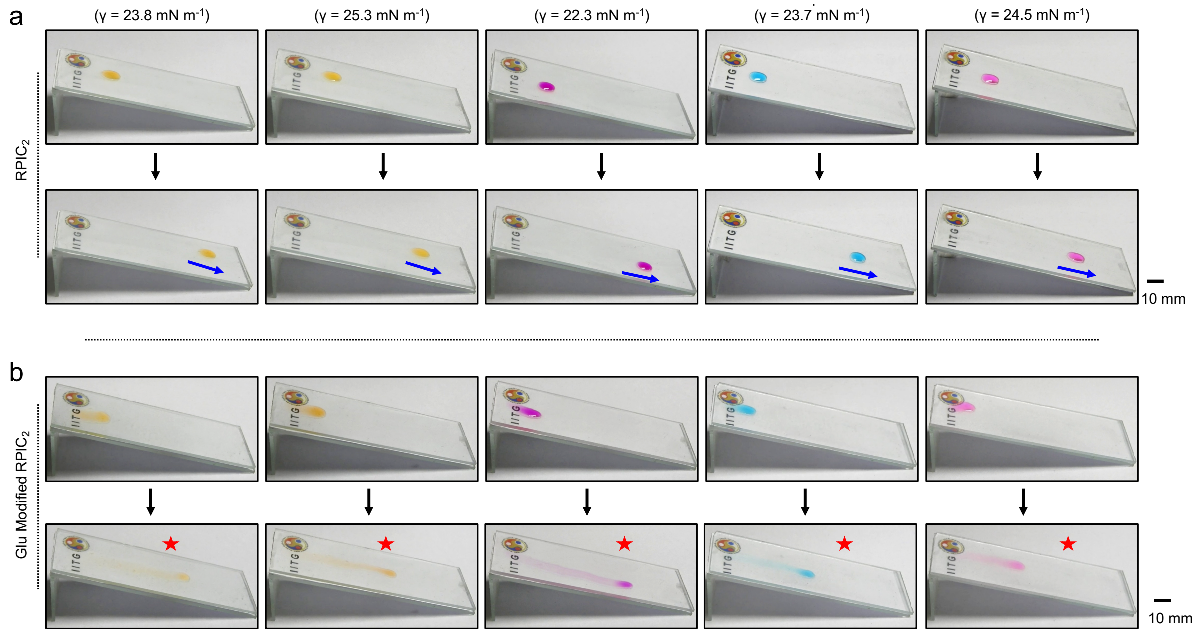

Supplementary Figure 16. Impact of glucamine modification of $RPIC_2$ on anti-wetting property for low surface tension liquids. **a** Digital images depicted the sliding of beaded droplets (volume 10 µl) of liquids having surface tension (γ) < 30 mN m^{-1} on tilted (15°) interface of reactive polymer infused coating-2 ($RPIC_2$). **b** Digital images depicted the arbitrary spillage of beaded droplets (volume 10 µl) of liquids having surface tension (γ) < 30 mN m^{-1} (volume 10 µl) on tilted (15°) interface of glucamine modified $RPIC_2$. Star mark indicates spillage of beaded droplets on solid surface.

Supplementary Note 17. The sliding of beaded droplets of high surface tension (> 35 mN m^{-1}) liquids were examined on both $RPIC_2$ and glucamine modified $RPIC_2$. Both the coatings displayed slippery behaviour towards liquids droplets having surface tension > 35 mN m^{-1}.

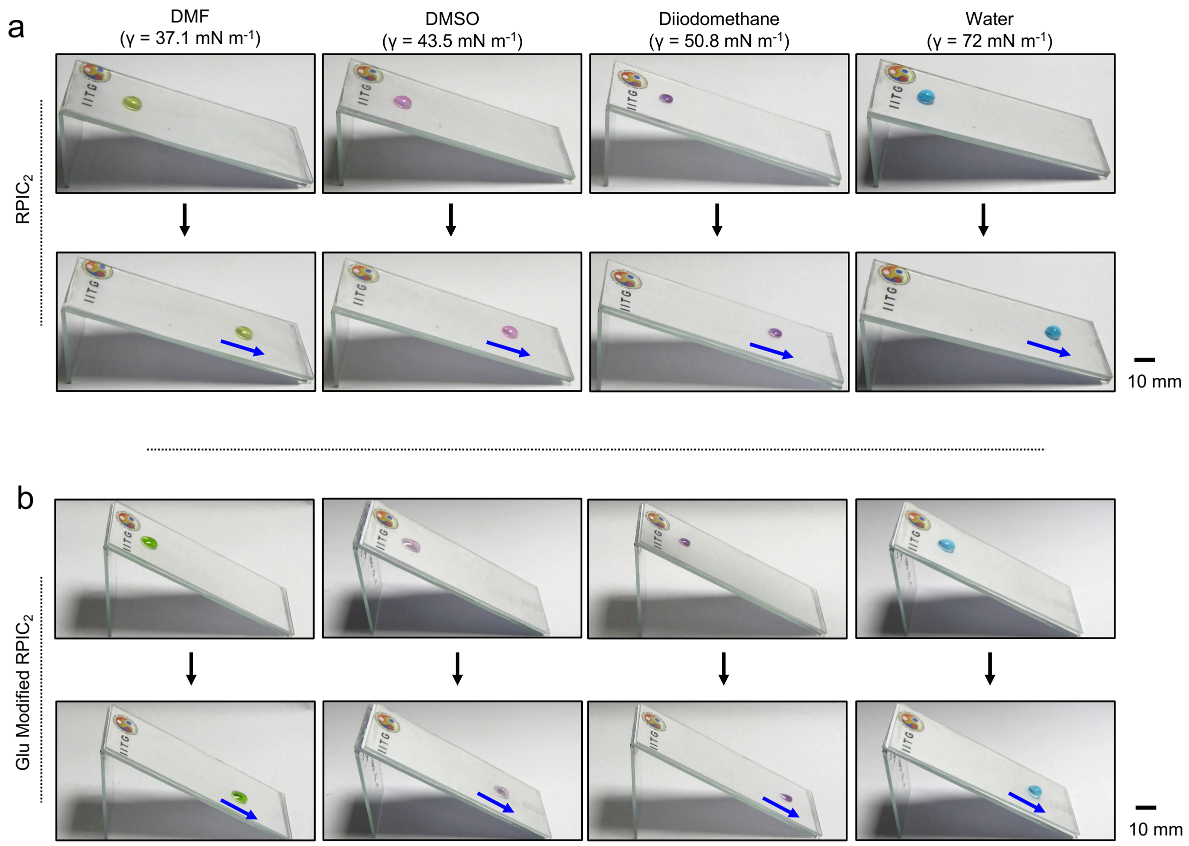

Supplementary Figure 17. Investigation of slippery property of $RPIC_2$ and glucamine modified $RPIC_2$ towards high surface tension liquids. a-b) Digital images depicted the sliding of beaded droplets of liquids having surface tension (γ) > 35 mNm^{-1} on tilted (20º) interface of $RPIC_2$ before (a) and after (b) glucamine modification.

Supplementary Note 18. Alteration in wettability of beaded non-polar and polar liquids after glucamine modification of $RPIC_2$ was characterized through static contact angle measurements. While droplets of low surface liquids suffered from spillage on glucamine modified $RPIC_2$, the droplets of high surface tension liquids continued to slide effortlessly.

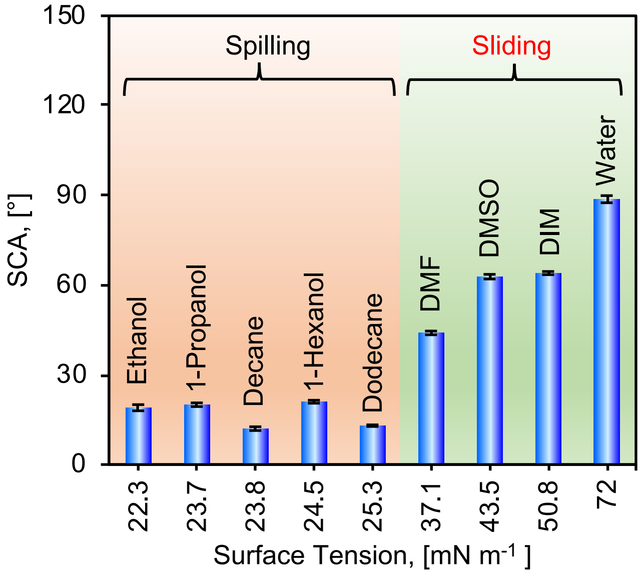

Supplementary Figure 18. Characterization of wettability of glucamine modified $RPIC_2$. Graph showing the selective alteration of anti-wetting property of glucamine-modified $RPIC_2$ against liquids having different surface tension. Orange and green highlighted region indicates spilling and sliding of beaded droplets on glucamine-modified $RPIC_2$, respectively. The error bar indicates the standard deviation with number of measurements, n=3 for each data point. Source data are provided as a Source Data file.

Supplementary Note 19. Glucamine modified $RPIC_2$ exhibited spillage towards low surface tension liquids (< 30 mN m^{-1}) over a wide range of tilting angles. This phenomenon was characterized by capturing digital images.

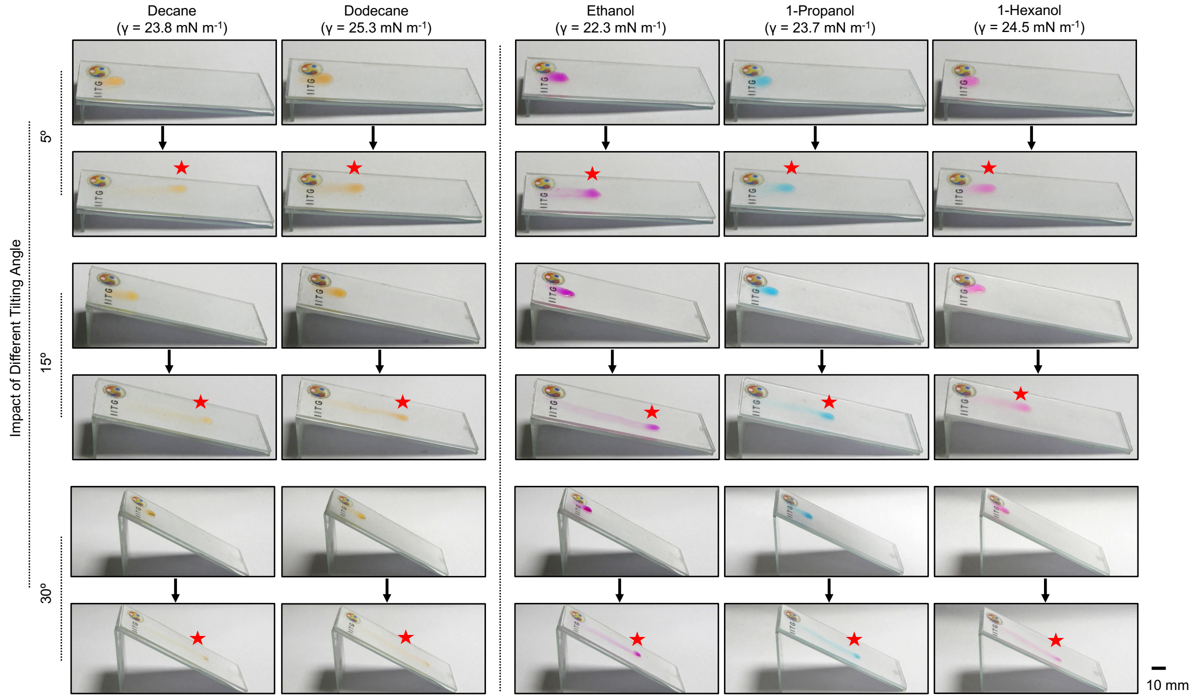

Supplementary Figure 19. **Spillage of low surface tension liquids independent of tilting angle.** Digital images illustrating the spillage of liquid droplets (volume 10 μl) having surface tension (γ) < 30 mN m^{-1} on glucamine modified $RPIC_2$ over a range of tilting angles from 5º to 30º. Star mark indicates spillage of beaded droplets on solid surface.

Supplementary Note 20. Glucamine modified $RPIC_2$ exhibited spillage towards low surface tension liquids (< 30 mN m^{-1}) irrespective of the volume of beaded liquids. This phenomenon was characterized by capturing digital images.

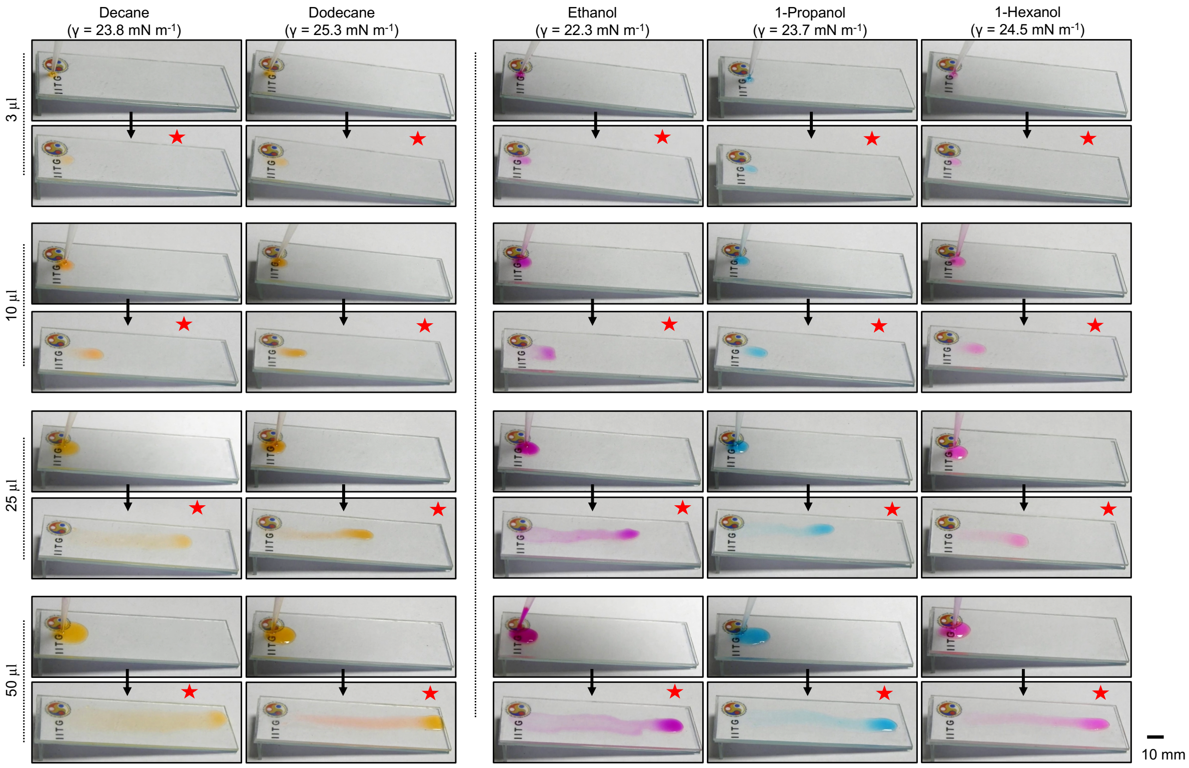

Supplementary Figure 20. Volume independent spillage of low surface tension liquids. Digital images illustrating the spillage of liquid droplets of different volumes (3 µl, 10 µl, 25 µl and 50 µl) having surface tension (γ) < 30 mN m^{-1} on the tilted interface (5°) of glucamine modified $RPIC_2$. Star mark indicates spillage of beaded droplets on solid surface.

Supplementary Note 21. Embedded slippery property of $RPIC_{PDAMA}$ altered significantly and selectively for low surface tension liquids and oil after glucamine modification. Glucamine modified $RPIC_{PDAMA}$ exhibited spillage towards low surface tension liquids (< 30 mN m^{-1}) and oils. This phenomenon was characterized by capturing digital images.

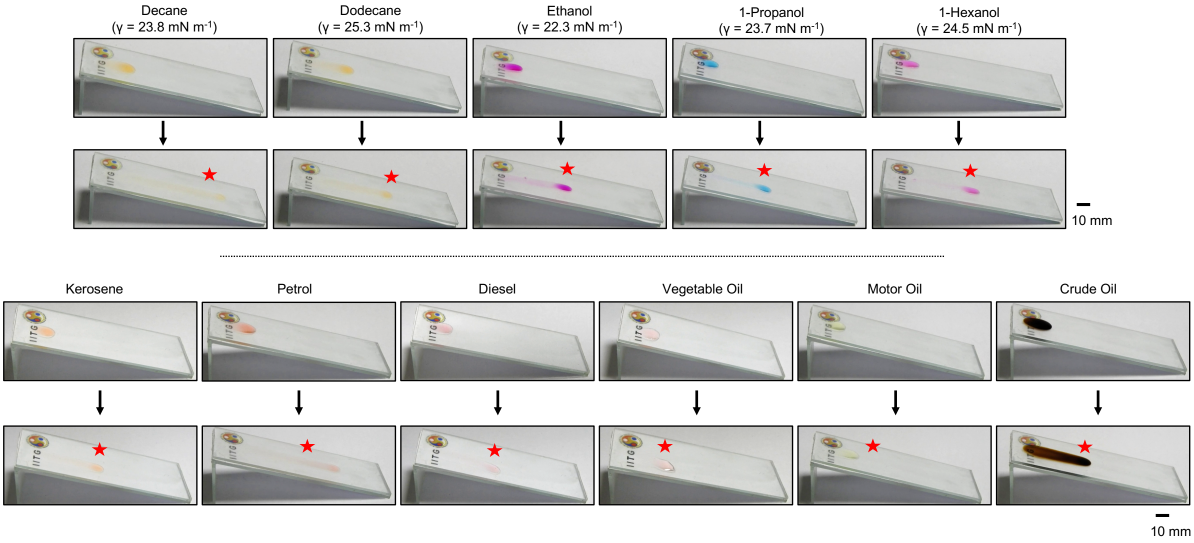

Supplementary Figure 21. **Spillages of low surface tension liquid droplets on glucamine-modified coating**. Digital images depicted the arbitrary spillage of beaded droplets (volume 10 µl) of organic solvents having surface tension (γ) < 30 mN m^{-1} and oils on tilted interface of glucamine modified coating ($RPIC_{PDAMA}$). Star mark indicates spillage of beaded droplets on solid surface.

Supplementary Note 22. Glucamine modification of $RPIC_{PDAMA}$ significantly altered its surface free energy (from ~ 20 mN m^{-1} to ~ 35 mN m^{-1}). However, this chemical modification of $RPIC_{PDAMA}$ merely perturbed the roughness (R_q) of the coating.

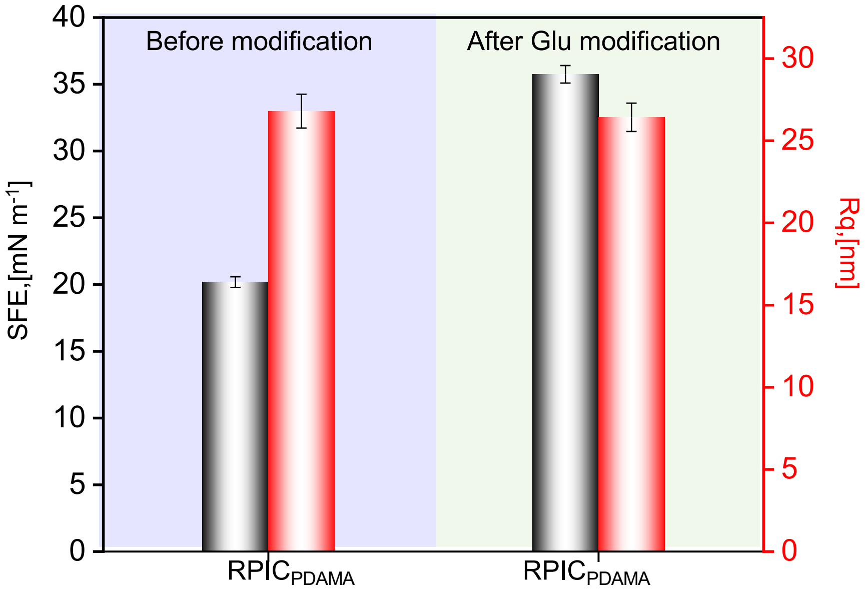

Supplementary Figure 22. Characterization of SFE and roughness of glucamine-modified $RPIC_{PDAMA}$. Plot accounting for the changes in surface free energy (SFE) and root mean square roughness (Rq) of unmodified and glucamine modified reactive polydocosyl acrylate maleic anhydride (PDAMA) infused coating ($RPIC_{PDAMA}$). Blue and green highlighted region indicates before and after modifications, respectively. The error bar indicates the standard deviation with number of measurements, n=3 for each data point. Source data are provided as a Source Data file.

Supplementary Note 23. The compromised anti-wetting property of glucamine modified $RPIC_2$ towards low surface tension liquids was restored by simply heating and followed by cooling down the coating to room temperature. This phenomenon was characterized by capturing digital images and measuring contact and sliding angles.

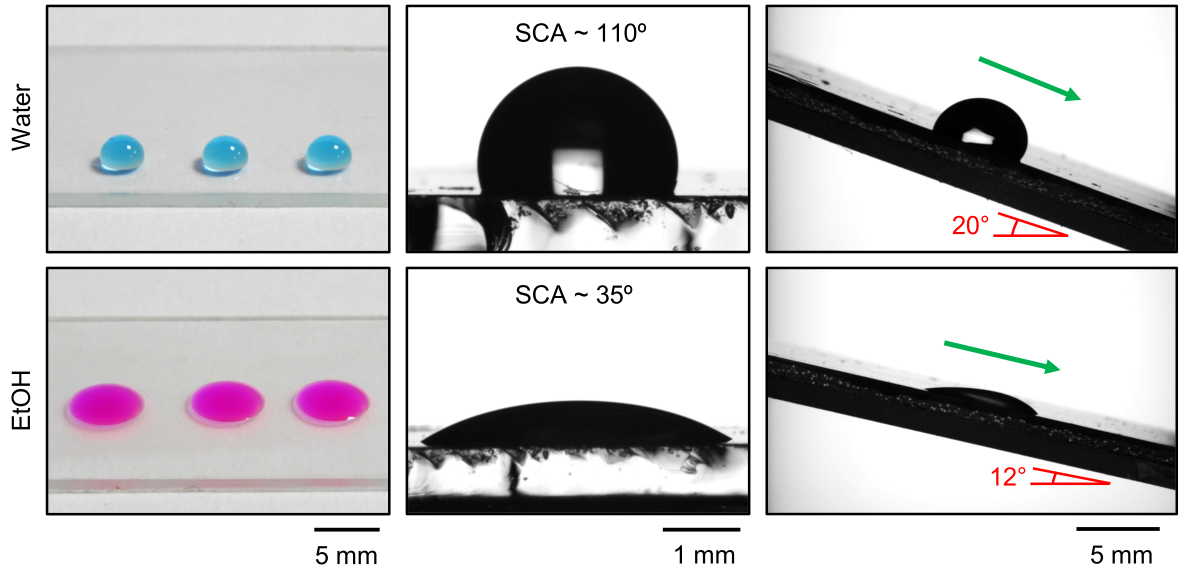

Supplementary Figure 23. Restoration of slippery property and wettability of $RPIC_2$ modified with glucamine via phase transition of infused polymer. Photographs and contact angle images showing the beading and sliding of water and ethanol droplets on glucamine-modified $RPIC_2$ after heating and subsequent cooling the interface.

Supplementary Note 24. The thickness of the coating ($RPIC_2$) was altered just by varying the loading amount of polymer in the porous matrix. The thickness of the coatings was determined using a profilometer.

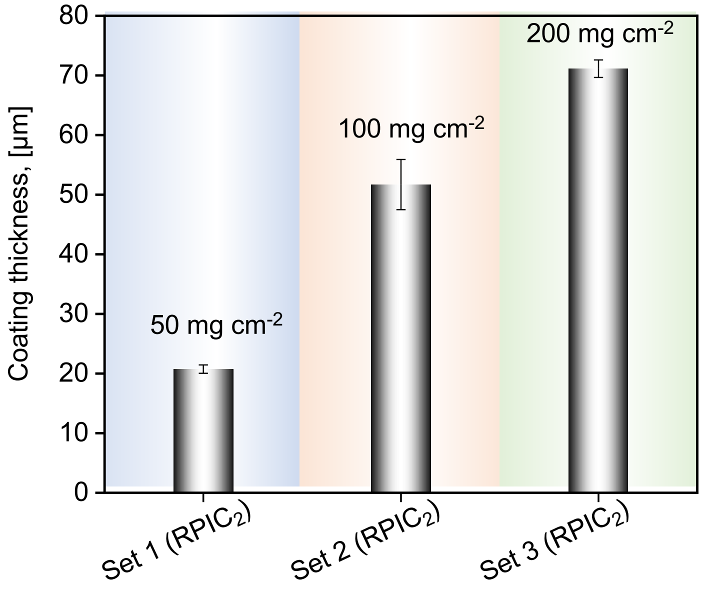

Supplementary Figure 24. Tailoring the thickness of $RPIC_2$. Plot accounting for the changes in coating thickness of reactive polymer infused coating-2 ($RPIC_2$) with increasing the loading amount of infused polymer polyoctadecyl acrylate maleic anhydride-2 ($PODAMA_2$) from 50 mg cm^{-2} to 200 mg cm^{-2}. Blue, orange, and green highlighted regions indicate the thickness of set 1, set 2, and set 3 coatings, respectively. The error bar indicates the standard deviation with number of measurements, n=3 for each data point. Source data are provided as a Source Data file.

Supplementary Note 25. The ability of reversible alteration of anti-wetting properties of $RPIC_2$ depends on the loading amount of infused polymer. The cyclic alternation of SCA and SFE was monitored for $RPIC_2$ having variation of loading amount of $PODAMA_2$.

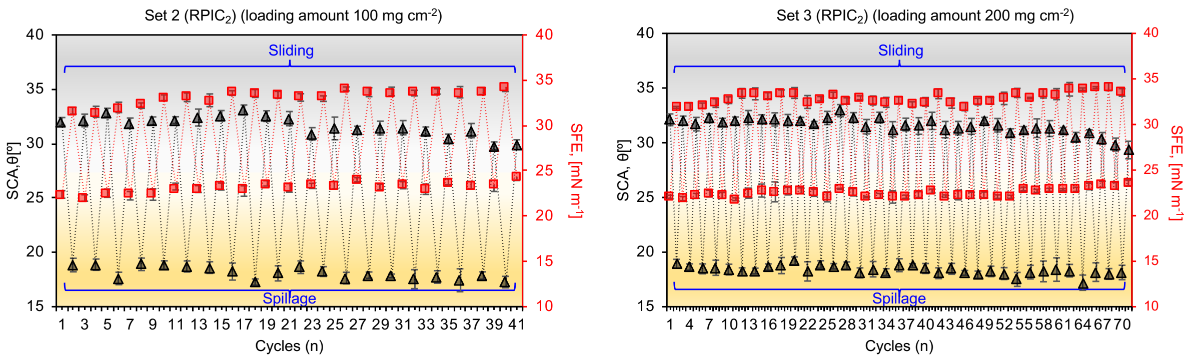

Supplementary Figure 25. Reversible alteration of anti-wetting property. Plot demonstrating the sliding and spillage of beaded droplet of ethanol on two distinct $RPIC_2$ having difference in loading amount of $PODAMA_2$, i.e. 100 mg cm^{-2} and 200 mg cm^{-2} for 40 and 70 cycles through repeating the glucamine modification and subsequently heat-treatment. Grey and yellow highlighted region indicates sliding and spillage, respectively. The error bar indicates the standard deviation with number of measurements, n=3 for each data point. Source data are provided as a Source Data file.

Supplementary Note 26. Low surface tension liquids (< 30 mN m^{-1}) recognized the glucamine modified path on $RPIC_2$ and travel from one end of the path toward the other end. However, the high surface tension liquids (> 35 mN m^{-1}) failed to recognize the glucamine-modified track—and followed a distinct path to reach other end of the patterned interface.

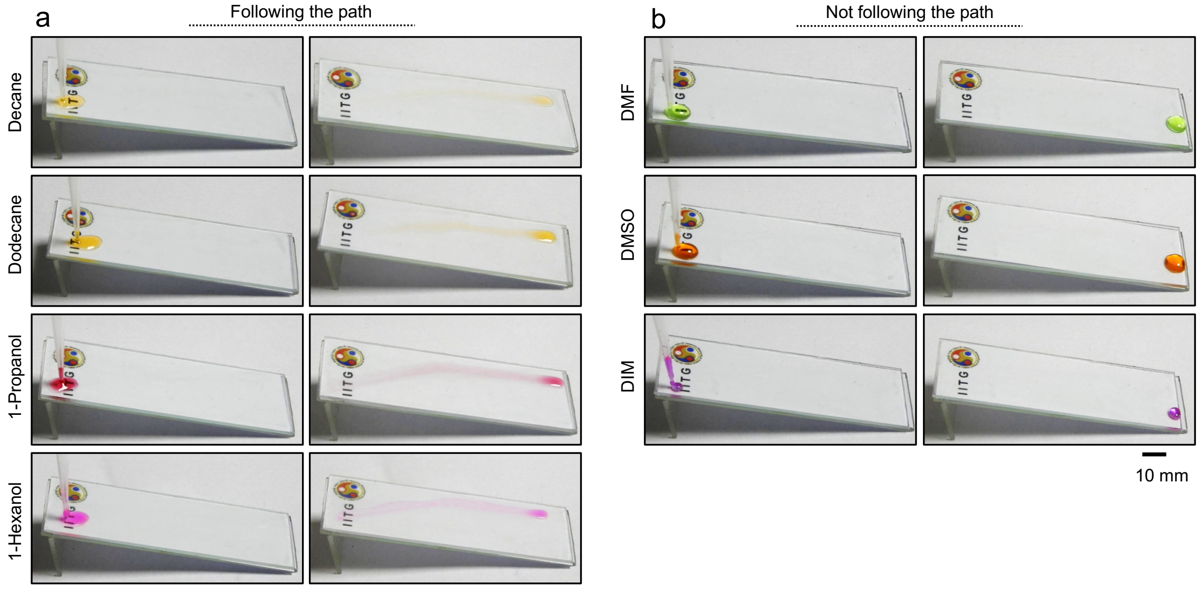

Supplementary Figure 26. Guided transport of low surface tension liquids. Photographs showing the guided transport of low surface tension liquids (a) (decane, dodecane, 1-propnanol, 1-hexanol) on the pattern interface, whereas high surface tension liquids (b) (dimethyl formamide (DMF), dimethyl sulfoxide (DMSO) and Diiodomethane (DIM)) failed to recognize the pattern—and followed a different path on the same interface to slide down.

Supplementary Note 27. Low surface tension liquid, i.e. ethanol recognized the glucamine modified path on $RPIC_2$ and travel from one end of the path toward the other end, even when the patterned interface kept with low tilting angle. However, even a larger volume of a beaded droplet (1 ml) of high surface tension liquid, i.e. water failed to recognize the glucamine modified track.

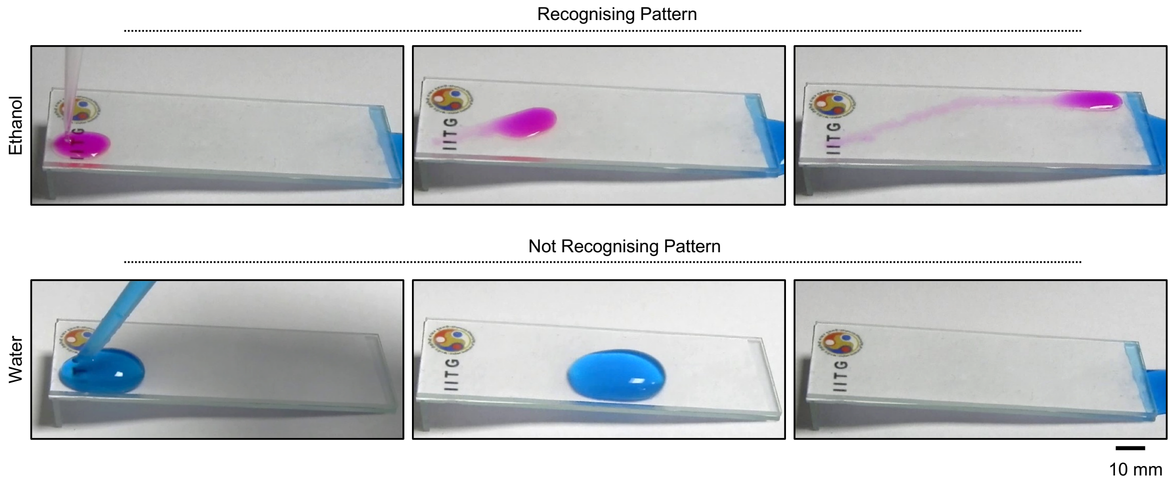

Supplementary Figure 27. Guided transport of ethanol at low tilting angle. Photographs showing the guided spilling of low surface tension liquid, i.e. ethanol alone the pattern interface, whereas high surface tension liquid, i.e. water failed to recognize the pattern—and effortlessly slide down following a different path on the same interface at a tilting angle of 5º.

Supplementary Note 28. Separation of water-hexanol mixture using glucamine modified patterned surface is performed and characterization using NMR spectroscopy. An array of glucamine-modified patterned spots readily captured hexanol (a low surface tension liquid) from water-hexanol mixture—and allowed the water droplet (a high surface tension liquid) to slide down to the other end of the pattern interface.

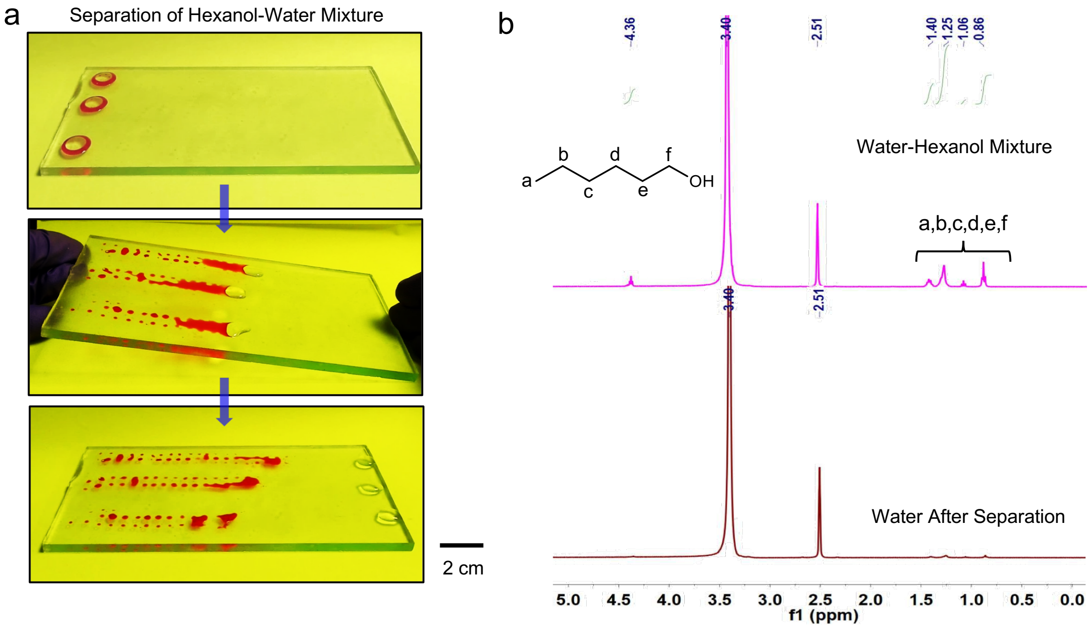

Supplementary Figure 28. **Separation of water-hexanol mixture by patterned interface.** **a** Photographs demonstrating the separation of water-hexanol droplets mixture on the patterned interface, where glu-modified patterned region selectively collected the hexanol droplets (dyed with Nile red)—and allowed water droplet to slide effortlessly. **b** NMR spectra of hexanol-water mixture before and after sliding on the pattern interface.
