# Supplementary material for: ‘Rewritable’ and ‘liquid-specific’ recognizable wettability pattern
Source: Nat Commun. 2024 Jul 11;15:5838. doi: 10.1038/s41467-024-49807-8 (PMC11239882; doi:10.1038/s41467-024-49807-8)
Supplement: Supplementary file 3 — Description of Additional Supplementary Files [file 41467_2024_49807_MOESM3_ESM.pdf]

Description of Additional Supplementary Files

File Name: Supplementary Movie 1
Description: This movie file is based on selective recognition of glucamine modified 'A' shaped patterned region by only low surface tension liquid ethanol, whereas the same pattern remains unrecognizable by high surface tension liquid water.

File Name: Supplementary Movie 2
Description: This movie file is based on sorting of liquid droplets based on their surface tension value. A wettability pattern was designed on the surface to track the movement of liquid droplets. The low surface tension liquid ethanol follows the patterned path, whereas high surface tension liquid water is unable to recognize the pattern and slides down in a different direction.

File Name: Supplementary Movie 3
Description: This movie file is based on the selective collection of oil droplet on the glucamine modified patterned region. During the movement of droplet mixture of water and crude oil, the patterned region selectively collects only crude oil, whereas oil-free water droplet travels away.
